# A Swollenin From *Talaromyces leycettanus* JCM12802 Enhances Cellulase Hydrolysis Toward Various Substrates

**DOI:** 10.3389/fmicb.2021.658096

**Published:** 2021-03-29

**Authors:** Honghai Zhang, Yuan Wang, Roman Brunecky, Bin Yao, Xiangming Xie, Fei Zheng, Huiying Luo

**Affiliations:** ^1^College of Biological Sciences and Biotechnology, Beijing Forestry University, Beijing, China; ^2^Institute of Animal Sciences, Chinese Academy of Agricultural Sciences, Beijing, China; ^3^Biosciences Center, National Renewable Energy Laboratory, Golden, CO, United States

**Keywords:** disruptive activity, synergistic effect, hydrolytic, biomass conversion, swollenin

## Abstract

Swollenins exist within some fungal species and are candidate accessory proteins for the biodegradation of cellulosic substrates. Here, we describe the identification of a swollenin gene, *Tlswo*, in *Talaromyces leycettanus* JCM12802. *Tlswo* was successfully expressed in both *Trichoderma reesei* and *Pichia pastoris*. Assay results indicate that *Tl*SWO is capable of releasing reducing sugars from lichenan, barley β-glucan, carboxymethyl cellulose sodium (CMC-Na) and laminarin. The specific activity of *Tl*SWO toward lichenan, barley β-glucan, carboxymethyl cellulose sodium (CMC-Na) and laminarin is 9.0 ± 0.100, 8.9 ± 0.100, 2.3 ± 0.002 and 0.79 ± 0.002 U/mg, respectively. Additionally, *Tl*SWO had disruptive activity on Avicel and a synergistic effect with cellobiohydrolases, increasing the activity on pretreated corn stover by up to 72.2%. The functional diversity of *Tl*SWO broadens its applicability in experimental settings, and indicating that it may be a promising candidate for future industrial applications.

## Introduction

Lignocellulosic biomass or plant dry matter, has been considered an alternative to fossil fuels. However, the growth and processing of biomass feedstocks for conversion into fuels, or for other chemical production purposes, remain challenging (Galbe and Wallberg, 2019). A successful pretreatment process is measured in two ways. Firstly, by the highly efficient recovery of carbohydrates from raw materials. Secondly, by minimizing the formation of toxic and inhibitive compounds to prevent unwanted health risks and environmental hazards (Bhatia et al., 2020). The enzymatic deconstruction of biomass that follows pretreatment can be enhanced using a number of non-glycoside hydrolase accessory proteins. These enzymes include, but are not limited to, expansins, loosenins, cerato-platanin proteins and certain other types of carbohydrate binding modules (CBM) (Gourlay et al., 2013; Payne et al., 2015; Guo et al., 2017; Luti et al., 2020). Among these proteins, expansins have been widely found in plant cell walls where they function to loosen the cellular wall (Marowa et al., 2016).

Mcqueen-Mason proposed that expansins can disrupt hydrogen bonding between plant cell wall polysaccharides without hydrolyzing them (Mcqueen-Mason and Cosgrove, 1994). In 2002, the first expansin-like protein SWOI from fungi was discovered in *Trichoderma reesei* (Saloheimo et al., 2002). Fungal swollenins have sequence similarity to expansins and are often referred to as expansin-like proteins. Indeed, SWOI was able to disrupt cotton fibers and filter paper structures on a microscopic level without detectable reducing sugars (Saloheimo et al., 2002). Over the past decade, more than 10 types of swollenins have been identified (Yao et al., 2008; Zhou et al., 2011; Kang et al., 2013; Santos et al., 2017) and the Expansin engineering Database (ExED^1^), has recently been released to the public (Lohoff et al., 2020). In general, expansins are no longer than 250 amino acids and have a two-domain structure. The primary domain of expansins resembles the glycoside hydrolase family 45 (GH45) and this homology preserves certain sequence features of the GH45 catalytic site (Bharadwaj et al., 2020). The second domain has a characteristic flat aromatic-rich surface and is homologous to group-2 grass pollen allergens. Some studies have proposed that this domain functions as a CBM (Cosgrove, 2000; Georgelis et al., 2012). Within the expansin, the two domains are interconnected by a short linker, and both domains are required for plant cell-wall loosening activity. However, the structural discrepancies between swollenins and expansins lead to functional differences. For example, swollenins have an additional CBM domain, making them homologous to fungal cellulases in the N-terminal (Eibinger et al., 2016). In other cellulases, CBMs direct the binding of the enzymes to cellulosic surfaces and enhance lignocellulose degradation (Hoffrén et al., 1995; Velikodvorskaia et al., 2013; Maharjan et al., 2018). Additionally, the O-glycosylation of linkers in swollenins may also have an effect on the enzyme binding to surfaces (Amore et al., 2017).

Previous studies have also described the synergy between swollenins and glycoside hydrolases in releasing soluble sugars from substrates. Zhou et al. successfully expressed swollenin SWO2 in *Aspergillus niger*, showing that the simultaneous incubation of SWO2 with cellulases results in a significant synergistic increase in cellulose hydrolysis activity. This synergy was further improved upon pretreatment of cellulose with swollenin (Zhou et al., 2011). Other investigations have focused on the synergistic relationship between swollenin and xylanase. Santos et al. (2017) found that the *Tl*SWO swollenin from *T. harzianum* creates a rough and amorphous surface on Avicel and has a highly synergistic effect in combination with a commercial xylanase from *T. viride*, enhancing its hydrolytic performance by up to 147%. Furthermore, Anthony et al. (2006) showed that a chimeric enzyme with *T. reesei* swollenin fused with *A. niger* feruloyl esterase A could significantly increase ferulic acid release from lignocellulose samples. Other studies have demonstrated that swollenins can release reducing sugars from cellulosic materials. For example, swollenin SWO2 from *T. pseudokoningii* and *Af*Swo1 from *Aspergillus fumigatus* exhibit very low levels of endoglucanase activity (Chen et al., 2010; Zhou et al., 2011). Recently, Andberg et al. (2015) demonstrated that swollenin SWOI from *T. reesei* had activity on substrates containing β-1,4 glycosidic bonds, and hypothesized a unique mode of mechanistic action with similarities to both endoglucanases and cellobiohydrolases (Andberg et al., 2015).

Previously, we identified the thermophilic *T. leycettanus* strain JCM12802 which is an excellent CAZyme source (Wang et al., 2015, 2016a,b; Xia et al., 2016). In this study, we present a swollenin protein, *Tl*SWO, that was cloned from *T. leycettanus* JCM12802, and successfully expressed in *T. reesei* and *Pichia pastoris*. We examined the *Tl*SWO enzymatic performance on various substrates and showed that *Tl*SWO can release reducing sugars from barley β-glucan, lichenan, laminarin and carboxymethyl cellulose sodium (CMC-Na). Additionally, we showed that *Tl*SWO can reduce Avicel particle size and surface structure, and significantly increase synergistic activity by up to 72.2% on pretreated corn stover (PCS).

## Materials and Methods

### Strains and Plasmids

*Talaromyces leycettanus* JCM12802, the donor strain, was purchased from Japan Collection of Microorganisms RIKEN BioResource Center (Tsukuba, Japan). *Escherichia coli Trans* I-T1 (TransGen, Beijing, China) was used for routine gene cloning. *T. reesei* AST1116 and *P. pastoris* GS115 (Invitrogen, Carlsbad, CA, United States) were used as hosts for gene expression. pPIC9 (Invitrogen) and pTrEno plasmids of were used to drive *Tlswo* gene expression in *P. pastoris* and in *T. reesei*, respectively. The pTrEno plasmid was constructed described by Linger et al. (2015).

### Sequence Analysis

*Tl*SWO DNA and amino acid sequences were analyzed using BLASTx and BLASTp programs^2^, respectively (Johnson et al., 2008). *Tl*SWO introns and exons were predicted using the GENSCAN Web Server^3^ (Burge and Karlin, 1997). SignalP 4.0 was used to predict the signal peptide sequence^4^ (Petersen et al., 2011). Potential N-glycosylation sites were predicted online^5^. Sequence assembly and estimation of the molecular mass and pI of the mature peptide were achieved using the Vector NTI Suite 10.0 software (Invitrogen). Protein molecular weight and molar extinction coefficients were estimated at the ExPASy tools page^6^. Multiple sequence alignments were performed using the Clustal W program from MEGA software 4.0. PROSITE^7^ was used to analyze protein domains and functional sites (Nicolas et al., 2006). The DiANNA web server^8^ was used to predict protein disulfide bond topology (Ferrè and Clote, 2005).

### Gene Cloning and Recombinant Protein Expression

*Talaromyces leycettanus* JCM12802 was cultured at 42°C for 3−5 days in the inducing medium (Wang et al., 2016c) with modifications. The medium contained 5.0 g/L (NH_4_)_2_SO_4_, 1.0 g/L KH_2_PO_4_, 0.5 g/L MgSO_4_⋅7H_2_O, 0.2 g/L CaCl_2_, 10.0 mg/L FeSO_4_⋅7H_2_O, 30.0 g/L wheat bran, 30.0 g/L soybean meal and 30.0 g/L corncob. *T. leycettanus* JCM12802 genomic DNA was extracted using the DNA isolation kit (Tiangen) following the manufacturer’s instructions and was used as a template for PCR amplification. Total RNA isolation and first strand cDNA synthesis were performed as previously described (Zhao et al., 2010). PCR amplification was performed using FastPfu DNA polymerase (TransGen). The *Tlswo* fragment corresponding to the 21–503 amino acid sequence was amplified using primers P1 (5′- GCTCGTGCTCAGAGCAGCTGTGCAGG-3′) and P2 (5′- CTAGATTACCTAGGTAAACTGCACC-3′). Amplified PCR products were cloned into pPIC9 and pTrEno vectors using Gibson Assembly (New England Biolabs, Ipswich, MA, United States) following the manufacturer’s protocol for cloning and expression. *Escherichia coli Trans* I-T1 (TransGen) used for routine gene cloning, was grown at 37°C overnight in Luria-Bertani medium supplemented with 50 μg/mL of ampicillin (Sigma-Aldrich, St. Louis, MO, United States).

When using *P. pastoris* GS115 as the expression host, pPIC9-*Tlswo* recombinant plasmids were linearized with *Bgl*II (New England Biolabs) and transformed into the expression host via electroporation. Positive transformants were screened on minimal dextrose medium at 30°C for 3 or 4 days until single colonies appeared. Single colonies were placed into shaking tubes for enzyme production using protocol provided in the *Pichia* Expression Kit (Invitrogen). Large-scale fermentation was performed as previously described (Zheng et al., 2018). The recombinant *P. pastoris* GS115 transformant containing pPIC9-*Tlswo* was grown at 30°C in 400 mL BMGY medium in a 1 L flask with shaking at 200 rpm for 48 h. Cells were collected and resuspended in 200 mL buffered methanol-complex (BMMY) medium with 0.5% (v/v) methanol and cultured at 30°C for 72 h with shaking (200 rpm). Methanol was added into the medium every 24 h.

When using *T. reesei* AST1116 as the expression host, recombinant pTrEno-*Tlswo* plasmids were linearized with *Sbf*I (New England Biolabs, United Kingdom) and used to transform *T. reesei* AST1116 via electroporation. Potato dextrose (PD) plates were used for spore production and PDHX plates (PD plates with hygromycin and TritonX-100 at final concentrations of 100 μg/mL and 0.1%, respectively) were used for screen potential *T. reesei* transformants which were grown for 2 to 3 days at 30°C. Mandels and Andreotti medium with 5% glucose (MAG) was used as the growth medium for *Tl*SWO expression. Subsequently, complete medium lactose (CML) was used for overexpression of the transformants. MAG and CML medium protocols were previously described by Linger et al. (2015). For large-scale fermentation, positive transformant spore stocks were streaked on potato dextrose agar plates and allowed to grow for 2 to 3 days until a well-developed plate of spores was formed. The wide end of a sterile 1.0-mL pipette tip was used to extract an approximately 0.5-cm plug from the plate and transferred into 1.0 L of MAG medium in a 2.8-L shake flask. The culture was grown at 28°C with 225 rpm shaking for 24 h, after which the entire 1.0 L was transferred to 7.0 L of the same medium in a bioreactor. The entirety of 8.0 L of medium were mixed at a 200 rpm and grown, after which a filtered air of 1.0 vol^∗^vol^–1*^min^–1^ was used to purge while the system was kept at a constant temperature of 28°C, and a pH of 4.8 for 48 h by using 2M KOH and HCl (Linger et al., 2015).

Then the culture broths were extracted for SDS-PAGE and activity assay analyses. Culture broths were clarified via centrifugation and transferred to microcentrifuge tubes. Broths were diluted 3:1 in 4 × LDS (Lithium dodecyl sulfate) sample buffer (Life Technologies Corp., Carlsbad, CA, United States) with 50 μL/mL β-mercaptoethanol as a reducing agent. Samples were incubated at 95°C for 5 min before loading onto NuPAGE SDS gels with MOPS buffer, and proteins were electrophoresed at 200 V for approximately 40 min.

### Protein Purification

Fermentation broths were harvested and sequentially vacuum-filtered. Filtered broth was then concentrated by tangential ultrafiltration with a 10 kDa MWCO (GE Healthcare, Chicago, IL, United States). The broths were roughly concentrated to volumes of 100 mL. The final concentrated volume was exchanged with at least 2.0 L of 20 mM Bis-Tris pH 6.5 to remove residual peptides and other low molecular weight debris. The following purification steps were then performed as previously described (Linger et al., 2015). The crude enzyme was purified through hydrophobic interaction chromatography (HIC) using a 26/10 Phenyl Sepharose Fast Flow column (GE Healthcare). Then the protein was subjected to anion exchange chromatography using a 10/100 anion exchange column packed with Source 15Q (GE Healthcare), HIC using a Source 15 iso 10/100 column (GE Healthcare), and size exclusion chromatography (SEC) using a 26/60 Superdex 75 column (GE Healthcare). The mobile phase was 20 mM acetate buffer pH 5.0, 100 mM NaCl.

Sodium dodecyl sulfate polyacrylamide gel electrophoresis (SDS-PAGE) was performed to assess purity of the *Tl*SWO protein. Proteins were separated on a 12% gel and visualized by Coomassie Blue staining. Protein concentration was measured using a NanoDrop 2000 Spectrophotometer (Thermo Fisher Scientific Inc., Rockford, IL, United States) and the Bradford protein assay kit (Bio-Rad).

### *Tl*SWO Activity Assays

*Tl*SWO activity was measured using the 3,5-dinitrosalicylic acid assay (Miller, 1959). Enzyme activity was assayed in a final volume of 1.5 mL, with 1% (w/v) barley β-glucan (Megazyme Co., Bray, Ireland), lichenan (Megazyme Co.), laminarin (Megazyme, Wicklow, Ireland) and carboxymethyl cellulose sodium (CMC-Na) (Sigma-Aldrich, St. Louis, MO, United States) as the substrates, and 10 μg/mL of enzyme at optimal conditions for 10 min. One unit of enzyme activity was defined as the amount of enzyme required to release 1 μmol of reducing sugars in 1 min.

### Effect of pH and Temperature on *Tl*SWO Activity

The effects of pH and temperature on *Tl*SWO activity were measured and compared. To determine the optimum pH of *Tl*SWO, enzyme activity was assayed using 1% lichenan (w/v) in a buffer of different pH, 100 mM glycine-HCl (pH 1.0–3.0), McIlvaine buffer (pH 3.0–8.0) and glycine–NaOH (pH 9.0–12.0). For pH stability, *Tl*SWO was preincubated at 37°C for 1 h in buffers of different pH (1.0–12.0) and subjected to the residual activity assay. The optimum temperature of *Tl*SWO activity was determined in a reaction solution of pH 4.0 using a temperature range from 30 to 80°C. The *Tl*SWO thermostability assay (100 μg/mL) was performed out by preincubating the enzyme at 37, 50, 60, or 70°C for 0–60 min, and performing residual activity assays on 100 μL aliquots withdrawn at different time points.

### Light and Scanning Electron Microscopic Analyses

Avicel PH-101 was used as a solid cellulosic substrate. Avicel (10 mg) was incubated with different amounts of purified *Tl*SWO in 100 mM citric acid-Na_2_HPO_4_ buffer (pH 4.0). The experiment was performed on a rotary shaker at 40°C for different time intervals. Control experiments without *Tl*SWO were also performed under the same conditions. The physical structure of Avicel fibers was initially observed using light microscopy (Olympus TH4-200, Japan). Subsequently, photomicrographs of the samples were captured using a scanning electron microscope (Hitachi SU8010, Tokyo, Japan) at a voltage of 15 kV.

### Polysaccharide Depolymerization Analysis

Hydrolysis reactions on 1% barley β-glucan, 1% CMC-Na, 1% laminarin and 0.5% lichenan, were performed overnight at pH 4.0 and 37°C using the 100 μg/mL of the enzyme. High-performance anion-exchange chromatography (Thermo Fisher Scientific, Sunnyvale, CA, United States) equipped with a Carbo-Pac PA200 column (3 × 250 mm) was used to determine the reaction products released from the polysaccharide.

### Synergism Between *Tl*SWO and Cellulases

The substrates used in this work were National Renewable Energy Laboratory (NREL) dilute acid PCS P120927, cellulose nanocrystals (CNCs) and phosphoric acid swollen cellulose (PASC). Each substrate was equivalent to 8.5 mg of glucan. For CNC preparations from Avicel, about 2 g of Avicel was added to HCl pre-heated at 80°C. Then, the acid hydrolysis was run for 4 h, and stirred every 15 min using a glass or Teflon rod, followed by centrifugation several times at 1,600 × *g* for 10 min. The supernatant was then decanted and the pellet resuspended in deionized (DI) water until the pH reached 5.0. CNCs were pelleted by centrifugation, collected and resuspended in DI water. Each substrate was suspended in 20 mM sodium acetate buffer, pH 5.0 and reactions were performed in triplicate in vials at 40°C. Avicel PASC was prepared as described by Zhang et al. (2006). The enzyme cocktail comprised endoglucanase I from *Trichoderma longibrachiatum* (Megazyme Co.), cellobiohydrolases Cel7A from *Penicillium funiculosum* and β-glucosidase from *Aspergillus niger* (Megazyme Co.) at concentrations (mg protein/g of glucan) of 2, 10, and 1, respectively. The reaction was carried out for 120 h, with sampling every 24 h. Samples of 100 μL, containing both solids and liquid were removed and diluted for HPLC sugar analysis using a Bio-Rad HPX-87H column. Control experiments using bovine serum albumin were also performed under the same conditions.

## Results

### Identification and Characterization of the *Tlswo* Gene

**FIGURE 1:**
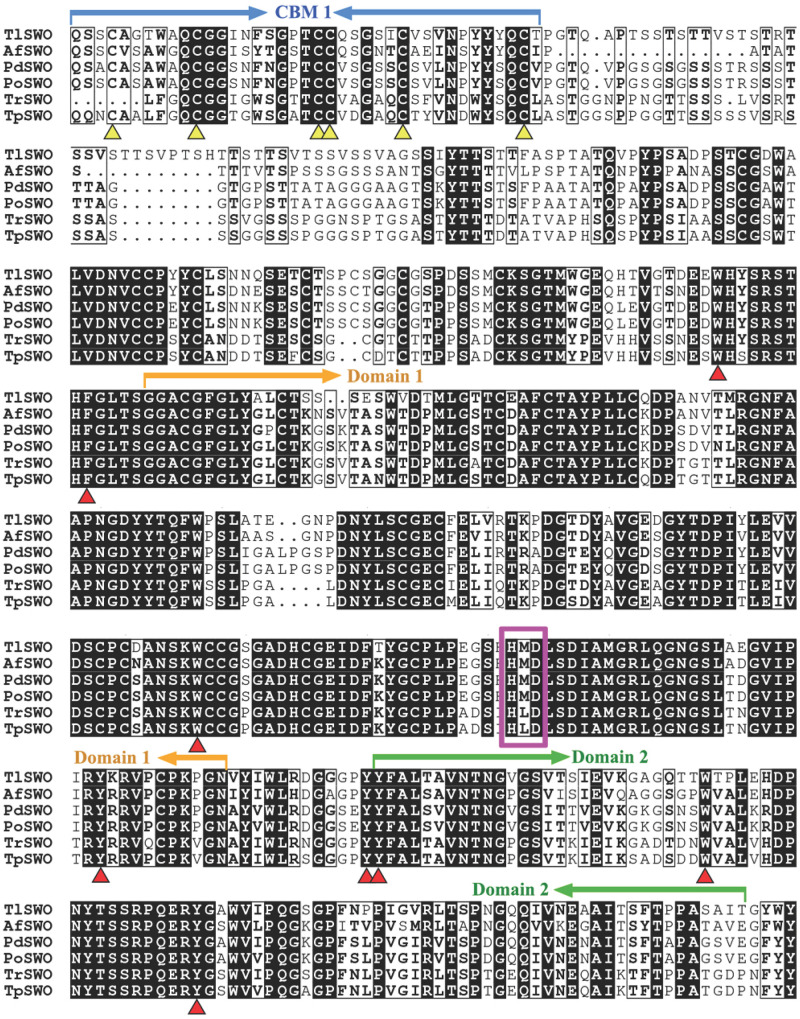
Sequence alignments of *Tl*SWO and five other swollenins from fungi. *Af*SWO from *Aspergillus fumigatus* (GeneBank No. XP_747748), *Pd*SWO from *Penicillium decumbens* (GeneBank No. ACH57439), *Po*SWO from *Penicillium oxalicum* (GeneBank No. ADZ74267), *Tr*SWO from *Trichoderma reesei* (GeneBank No. CAB92328) and *Tp*SWO from *Trichoderma pseudokoningii* (GeneBank No. ABV57767.1).

The putative *Tls*wo open reading frame consists of six exons and encodes a 503 amino acid protein (*Tl*SWO) and a signal peptide at the cleavage site between amino acids 20 and 21. The cloned gene sequence encoding a putative *Tl*SWO was submitted to NCBI GenBank as MT180127. Deduced *Tl*SWO shared 71.5% sequence similarity to the swollenin (BAI83433.1) from *A. fumigatus*, 71.2% to the swollenin (ADZ74267.1) from *P. oxalicum*. Further analysis using PROSITE demonstrated that *Tl*SWO consists of three domains, fungal-type carbohydrate-binding module family 1 (CBM1) (amino acids 23−59), family 45 endoglucanase-like domain of expansin (Expansin_EG45) (amino acids 206−388) and a cellulose-binding-like domain of expansin (Expansin_CBD) (amino acids 400−492), all of which are typical of swollenins from fungi (Figure 1). The six cysteines in the CBM1 of *Tl*SWO were highly conserved. DiANNA disulfide bond prediction identified three disulfide bonds in the CBM1 of *Tl*SWO (Cys4-Cys21, Cys11-Cys28, and Cys22-Cys28). CBM1 and Expansin_EG45 are connected by a Serine-Threonine rich linker domain. Although the function of the linker has been studied in other cellulases, it is not clear if the linker plays a similar role in swollenins (Kolaczkowski et al., 2020; Nakamura et al., 2020; Pena et al., 2020). Sequence alignment of swollenins revealed that *Tl*SWO maintained the conserved HMD (histidine, methionine, aspartic acid) catalytic motif of the GH45 cellulase (HFD, histidine, phenylalanine, aspartic acid), which is part of the active site (Figure 1). The aspartic acid of GH45 HFD is the proton donor during the catalytic process. However, the other residue of the catalytic active site, aspartic acid, is absent from both swollenins and some GH45 cellulases. The expansin CBD in the *Tl*SWO C-terminal region is homologous to the pollen allergen. There are also several conserved aromatic amino acids in the *Tl*SWO sequence, including Y400, Y401, F402, W429, Y447, W450, Y496, and F503, which may play key roles in substrate binding (Figure 1).

### Expression of *Tl*SWO in *P. pastoris* GS115 and *T. reesei* AST1116

*Tl*SWO (483 amino acids, theoretical molecular weight 51.1 kDa) was expressed in *P. pastoris* and *T. reesei* using the *aox1* and *eno* promoters, respectively (Supplementary Figure 1). Based on the results of SDS-PAGE (Figure S1), the quantity of *Tl*SWO expressed was higher in *T. reesei*, thus all the *Tl*SWO characterized in this study was expressed and purified from *T. reesei*. The purified swollenin protein migrated as a protein of ∼80 kDa. This single band was analyzed by MALDI-TOF MS because of the difference between the observed and predicted protein size, and the trypsin-digested peptide sequences were matched to the deduced *Tl*SWO amino acid sequence (Supplementary Figure 2). Sequence prediction results indicate that *Tl*SWO has five N-glycan sites (Asn35, Asn154, Asn249, Asn366, and Asn436). After Endo H digestion, the molecular weight of *Tl*SWO decreased to ∼72 kDa, which is still higher than theoretical MW (Supplementary Figure 1A). We speculate that the remainder of the molecular weight increase was caused by heavy O-glycan glycosylation in the linker region which is rich in serines and threonines, as was reported for other proteins expressed in these hosts (Bai et al., 2019).

### *Tl*SWO Activity on Different Substrates

**FIGURE 2:**
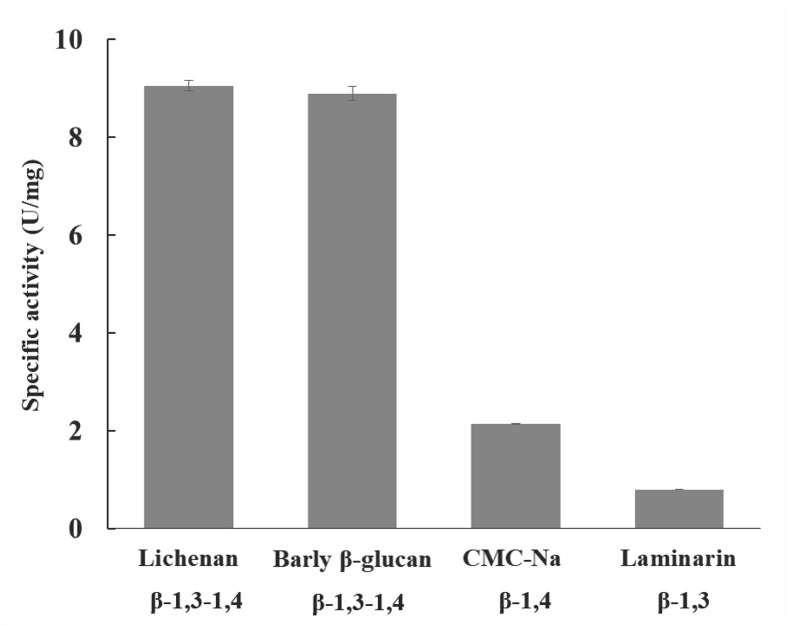
*Tl*SWO substrate specificity. Hydrolysis reactions using 1% barley β-glucan, 1% CMC-Na, 1% laminarin and 0.5% lichenan substrates were performed overnight at pH 4.0 and 50°C. The linkage type of each substrate is labeled.

*Tl*SWO cellulolytic activity was measured using the substrates lichenan, barley β-glucan, CMC-Na, laminarin, Avicel and glucomannan. Xylanase activity was measured with birchwood xylan, and mannase activity was measured with locust bean gum. All reactions were carried out overnight. Our results show that *Tl*SWO has significant activity on lichenan, barley β-glucan, glucomannan and CMC-Na, and a very low activity on laminarin. *Tl*SWO showed the highest activity on lichenan (9.0 ± 0.100 U/mg) and barley β-glucan (8.9 ± 0.100 U/mg), followed by CMC-Na (2.3 ± 0.002 U/mg). In contrast, a very low level of activity was observed with laminarin substrate (0.79 ± 0.002 U/mg) (Figure 2). Together, these results suggest that *Tl*SWO mainly acts on cellulose rich substrates and shows a preference toward substrates with 1,4 linkages.

### Effect of Temperature and pH on *Tl*SWO

**FIGURE 3:**
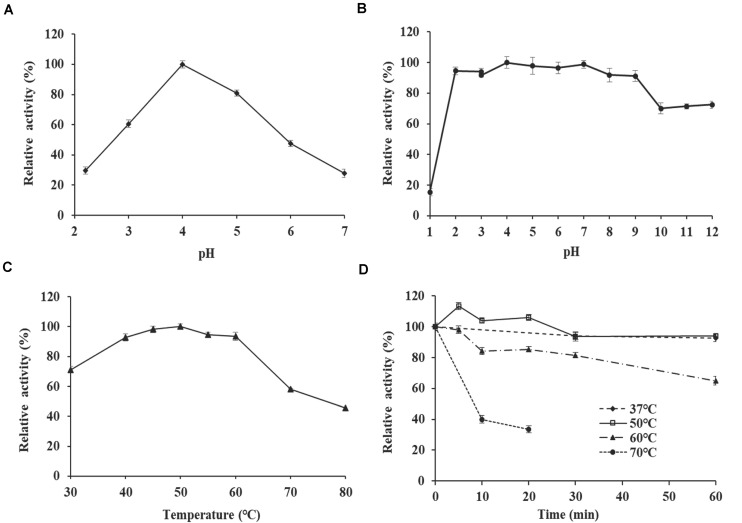
Enzyme properties of purified recombinant *Tl*SWO. **(A)** pH-activity profile tested at 50°C. **(B)** pH stability. After incubation of *Tl*SWO at 37°C for 1 h in buffers with pH levels ranging from 1.0 to 12.0, residual enzyme activity was determined at pH 4.0 and 50°C. **(C)** Temperature-activity profile tested at pH 4.0. **(D)** Thermostability. *Tl*SWO was pre-incubated at 37, 50, 60, and 70°C for different periods of time then subjected to the residual activity assay under optimal conditions.

The effect of pH and temperature on *Tl*SWO activity were investigated with lichenan as a substrate. Although the enzyme displayed activity across a broad pH range (2.0–12.0), we determined that the optimal pH for *Tl*SWO is 4.0 (Figure 3A). *Tl*SWO retained more than 80% of its activity within the pH range 2.0–9.0 after incubation at 37°C for 1 h. In contrast, *Tl*SWO lost 30% of its activity after incubation for 1 h at 37°C and pH 10.0–12.0 (Figure 3B). Additionally, *Tl*SWO reached optimal activity at 50°C and retained more than 90% of its activity within the temperature range of 40–60°C. However, after heating to 70°C or above, *Tl*SWO activity falls off very rapidly (Figure 3C). Additionally, *Tl*SWO maintained stable activity between 37 and 50°C after 1 h of incubation, but its activity decreased to 40% after 10-min at 70°C (Figure 3D).

### *Tl*SWO Mode of Action

**FIGURE 4:**
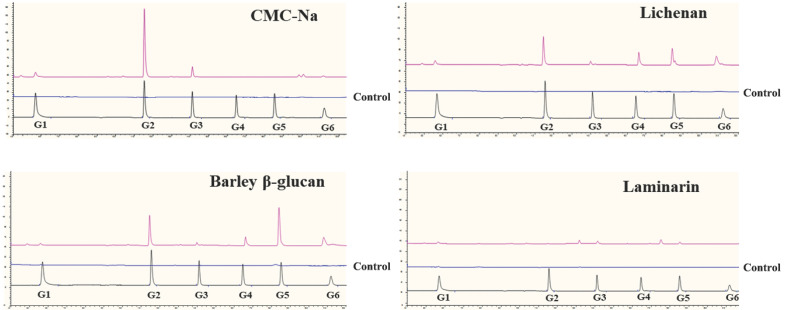
Hydrolysis capacity of *Tl*SWO to degrade CMC-Na, lichenan, barley β-glucan and laminarin. All reactions were performed overnight at pH 4.0 at 37°C using the enzyme in a final concentration of 100 μg/mL.

The *Tl*SWO mode of action was assessed using lichenan, barley β-glucan, glucomannan and CMC-Na (Figure 4). As a result, CMC-Na was hydrolyzed into cellobiose and a small amount of cellotriose (Figure 4). Analysis of the hydrolysis products of lichenan and barley β-glucan showed that *Tl*SWO preferentially hydrolyzed these two substrates into products with different degrees of polymerization, including cellobiose and cellopentose, followed by cellohexose and cellotetrose (Figure 4). We detected no sugar release after *Tl*SWO incubation with laminarin (Figure 4). These results suggest that *Tl*SWO may function as an endo-cellulase.

### Disruptive Action of *Tl*SWO on Avicel

**FIGURE 5:**
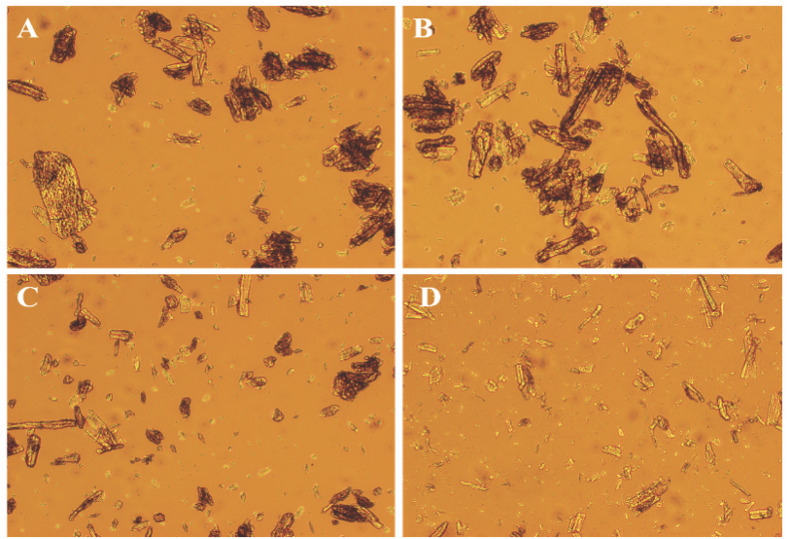
Light microscopic analyses of Avicel. Ten milligrams of Avicel was incubated with different amounts of purified *Tl*SWO in 100 mM citric acid-Na_2_HPO_4_ buffer (pH 4.0) for 24 h. **(A)** 0 μg *Tl*SWO **(B)** 10 μg *Tl*SWO, **(C)** 100 μg *Tl*SWO and **(D)** 300 μg *Tl*SWO.

The disruptive effect of *Tl*SWO on Avicel was evaluated using light microscopy (LM) and scanning electron microscopy (SEM). LM analysis showed that after incubation with different amounts of *Tl*SWO for 24 h, the Avicel’s physical structure significantly differed from that of untreated Avicel (Figure 5). Avicel was disrupted into smaller particles with increasing amounts of *Tl*SWO. Avicel pretreated with 300 μg of *Tl*SWO for 12 h was subjected to further analysis using SEM. In this sample, *Tl*SWO created a rough surface on Avicel when compared with the unpretreated sample (Supplementary Figure 3).

### Synergism Between *Tl*SWO and Cellulases

To test the capacity of *Tl*SWO in enhancing biomass hydrolysis via an enzymatic cocktail, we hydrolyzed pretreated biomass using cellulases alone first, followed by treatment using both cellulases and *Tl*SWO. Biomass degradation experiments were performed using β-glucosidase (EC 3.2.1.21), cellobiohydrolase (EC 3.2.1.91) and endoglucanase (EC 3.2.1.4) in the presence of *Tl*SWO. Reactions with BSA and without *Tl*SWO were used as controls. A total of 13 mg protein/g of glucan was used in all reactions. Endoglucanases randomly cleave internal β-1,4-glycosidic bonds to create new reducing ends. This allows cellobiohydrolases to continuously act on the chain termini to release cellobiose, and β-glucosidase then hydrolyzes cellobiose into glucose (Zheng et al., 2018). Therefore, the production of glucose, as the endpoint, was compared in the different reactions.

**FIGURE 6:**
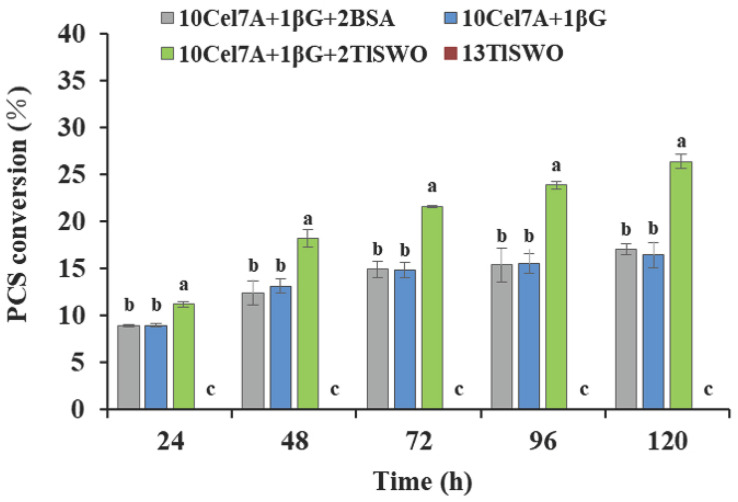
*Tl*SWO conversion performance on pre-treated corn stover (PCS). PCS hydrolysis performed at 40°C. Cellobiohydrolases Cel7A from *Penicillium funiculosum* and β-glucosidase from *Aspergillus niger* (Megazyme Co.) at concentrations (mg protein/g of glucan) of 13 and 1, respectively.

When using PCS as the substrate, *Tl*SWO exhibited significant synergetic effects in the presence of cellobiohydrolase Cel7A. No glucose was detected when PCS was reacted with *Tl*SWO at 13 mg protein/g of glucan, suggesting that PCS could not be hydrolyzed to monomers by *Tl*SWO alone (Figure 6). When the reaction contained Cel7A and β-glucosidase individually, PCS conversion increased from 8.9% at 24 h to 16.4% after 120 h. When Cel7A and β-glucosidase were supplemented with *Tl*SWO at 2 mg protein/g of glucan, glucose increased from 11.2% at 24 h to 26.4% at 120 h. Although *Tl*SWO alone did not produce detectable levels of released glucose, it significantly enhanced hydrolytic activity when added to Cel7A and β-glucosidase.

**FIGURE 7:**
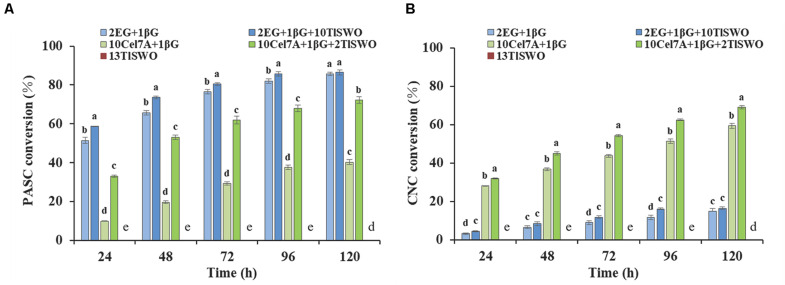
*Tl*SWO conversion performance. **(A)** Phosphoric acid swollen cellulose (PASC) hydrolysis and **(B)** cellulose nanocrystals (CNC) hydrolysis. All reactions were performed at 40°C. Endoglucanase I from *Trichoderma longibrachiatum* (Megazyme Co.), cellobiohydrolases Cel7A from *Penicillium funiculosum* and β-glucosidase from *Aspergillus niger* (Megazyme Co.) at concentrations (mg protein/g of glucan) of 2, 10, and 1, respectively.

Phosphoric acid swollen cellulose and cellulose nanocrystals exist in amorphous and crystalline forms, respectively, which may affect their binding with *Tl*SWO. Therefore, the effect of *Tl*SWO on these substrates was further examined. Similar to what we observed with PCS, *Tl*SWO could not release any sugars from PASC and CNC without the presence of other cellulase/s (Figure 7). When Cel7A and β-glucosidase were utilized, the PASC conversion rate increased from 9.9% at 24 h, to 40.2% at 120 h (Figure 7A). However, when Cel7A and β-glucosidase were supplemented with *Tl*SWO, the conversion rates increased from 33.0% at 24 h to 72.2% at 120 h. The 120-h data shows that the conversion rate of PASC increased by approximately 32% following *Tl*SWO supplementation, suggesting that *Tl*SWO has significant synergetic effects with Cel7A. Additionally, the synergetic effects of *Tl*SWO and endoglucanases were also explored. When using endoglucanase and β-glucosidase individually, the conversion rate increase was 51.4% at 24 h and 85.6% at 120 h. When used in combination with *Tl*SWO, the conversion rate was 58.6% at 24 h and 85.7% at 120 h. Although these conversion rates are slightly higher than those observed using endoglucanase and β-glucosidase individually, the conversion rate at 120 h was 86.4% and was within the margin of error when compared to 85.6%. Therefore, we conclude that the addition of *Tl*SWO did not significantly increase PASC enzymatic hydrolysis when used in combination with endoglucanase.

The cellulose conversion rate of CNC was overall significantly lower than that of PASC. Using CNC as a substrate, Cel7A and β-glucosidase achieved a conversion rate of 28.2% at 24 h and 59.3% at 120 h (Figure 7B), which increased further when *Tl*SWO was added. The glucose yields obtained by the enzyme cocktail systems containing *Tl*SWO, Cel7A and β-glucosidase were 31.9% at 24 h and 68.9% at 120 h, which is higher than all other cases when compared with the control group. Therefore, we conclude that *Tl*SWO has a synergistic effect with processive cellobiohydrolases. However, when used in combination with endoglucanases, *Tl*SWO does not produce any significant synergistic effect. After 120 h of enzymatic hydrolysis, the glucose yield released from CNC was 16.4% with the combination of *Tl*SWO, endoglucanase and β-glucosidase. This corresponds well with the hydrolysis rate of 14.9% observed using endoglucanase and β-glucosidase individually. Taken together, our results indicate that, *Tl*SWO acts more efficiently on amorphous cellulose than on the respective crystalline forms.

## Discussion

Several previous studies have shown that SWOI can disrupt plant cell wall structures without leaving any traceable amounts of reducing sugars. However, subsequent research has confirmed that SWOI does exhibit some hydrolytic activity on cellulosic substrates with features of both endoglucanases and cellobiohydrolases (Saloheimo et al., 2002; Andberg et al., 2015). Similar to results observed for SWOI, the other two swollenins, *Af*SWO1 from *A. fumigatus* and SWO2 from *T. pseudokoningii* have shown hydrolytic activity on various substrates, suggesting that these proteins interact with cellulose or hemicellulose (Chen et al., 2010; Zhou et al., 2011). In this study, *Tl*SWO from *T. leycettanus* JCM12802 was found to have similar functionalities as other fungal swollenins. *Tl*SWO was also shown to share relatively high sequence identity with SWOI, *Af*SWO1 and SWO2 (64.5, 73.7, and 63.2%, respectively). *Tl*SWO has the highest activity on lichenan and barley β-glucan substrates, both of which contain β-1,4 and β-1,3 linkages, and its activity is minimal on laminarin, a substrate that only contains β-1,3 linkages. This indicates that the primary mechanism of action of TlSWO is via activity on β-1,4 linkages. Expansins are more highly similar to GH45 subfamily C enzymes than to other members of the GH45 family (Igarashi et al., 2008; Godoy et al., 2018). These expansins have a HFD motif termed as part of their active site, and this motif is also present in *Tl*SWO (HMD). The aspartic acid in this motif plays the role of proton donor in GH45. Nevertheless, other key residues that are critical for catalytic activities are absent in both expansins and swollenins. This difference suggests expansins and swollenins may use an inverted mechanism during the catalytic process. In 2015, Nakamura et al. (2015) proposed that *Pc*Cel45A, which belongs to GH45 subfamily C, uses an imidic acid form of asparagine residue as a general base in the “Newton’s cradle” proton relay catalytic mechanism. This proposal sheds light on some potential mechanistic properties of expansin’s catalytic process. Horizontal gene transfer drives sequence differences between fungal swollenins and plant/bacterial expansins (Nikolas et al., 2014). These differences could mean that the inverted catalytic mechanism theory of *Pc*Cel45A is unfeasible when applied to fungal swollenins.

Fungal swollenins are roughly twice as large as plant and bacterial swollenins because their D1 and D2 domains contain extra sequence insertions including the additional N-terminal CBM with linkers. CBMs can increase the concentration of their parent enzyme substrate surface, leading to more rapid polysaccharide degradation (Bolam et al., 1998; Janne et al., 2003). Primary amino acid sequence analysis using BLAST indicates that *Tl*SWO contains an N-terminal CBM region (amino acid residues 21–59) that shows the highest similarity toward fungal GH6 family 1 CBMs. Six typical conserved cysteines present in the sequence may form three pairs of disulfide bonds. The *Tl*SWO CBM also contains three conserved aromatic residues (Trp28, Tyr54, and Tyr55), which are typical in GH6 and GH7 cellulase CBMs. These sequences are important for cellulase stability and activity during the reaction. Moreover, the linker length is crucial for cellulase activity (Srisodsuk et al., 1993). The linker region of *Tl*SWO is over 140 amino acids, and is longer than most reported swollenins and fungal cellulases (Sammond et al., 2012). Although CBMs and linkers have been well studied in cellulases, little is known about the role of these two regions in swollenins.

We have shown that when treated with *Tl*SWO, the smooth surface structure of the microcrystalline Avicel transitions into a rough texture. This is consistent with previous studies suggesting that swollenin proteins function to modify the cell wall (Cosgrove, 2017; Javier and Daniel, 2005). Previous studies have also shown that swollenins can synergize with other enzymes including cellulases and xylanases (Chen et al., 2010; Kang et al., 2013; Santos et al., 2017). In this study, we explored the ability of *Tl*SWO in boosting cellulosic substrate hydrolysis by different enzymes. When the cellulose was incubated with *Tl*SWO and cellobiohydrolases, a greater increase in glucose yields was observed. However, we observed no significant synergistic effect between *Tl*SWO and endoglucanases. These results differ from those of a previous report, in which swollenin exhibited strong synergistic interaction with endoglucanases (Gourlay et al., 2013). Therefore, we propose that *Tl*SWO has better synergistic activity with cellobiohydrolases. Cellulose degradation is summarized by the classical C_1_-C_*x*_ model (C_1_: non-hydrolytic components, C_*x*_: endo- or exo-acting cellulases). Using this model, one proposal hypothesizes that C_1_ can disrupt cellulose by displacing hydrogen bonds in the microfibril, leading to a more available structure for C_*x*_ (Liu and King, 1967; Payne et al., 2015). Eibinger et al., and Kang et al., speculate that swollenins, much like endoglucanases, can act as C_1_ components because of their disruptive activity in the enzymatic saccharification of lignocellulosic substrates. However, the mode of action of various C_1_ proteins needs to be further explored (Reese et al., 1950; Kang et al., 2013; Eibinger et al., 2016). Our results using PASC and CNC substrates with or without *Tl*SWO were used to compare the effects of swollenin on different crystallinity materials. We showed that the total glucose concentration increased by 32% when PASC was incubated with *Tl*SWO and cellobiohydrolases, compared to incubating with only cellobiohydrolases. However, when using CNC as the substrate, no significant difference between the groups with or without *Tl*SWO was observed. These results suggest that swollenin has a greater ability to bind and disrupt amorphous cellulose than it does crystalline cellulose. This may be due to the endoglucanase-like activity of *Tl*SWO, as demonstrated on the model substrates we previously used, or because it is able to bind amorphous substrates more easily than crystalline substrates due to its CBM.

## Conclusion

Here, we report that *Tl*SWO, from *T. leycettanus* JCM12802, is an acidic and mesophilic swollenin that has activity toward lichenan, barley β-glucan, carboxymethyl cellulose sodium and laminarin. A greater increase in glucose yield was observed when cellulose substrates were incubated with *Tl*SWO and cellobiohydrolases. Moreover, *Tl*SWO exhibited synergetic effects on cellobiohydrolase when using PCS and PASC as substrates. However, no significant synergistic effect was observed between *Tl*SWO and endoglucanases, suggesting that *Tl*SWO has better coordination with cellobiohydrolases.

Compared to chemical pretreatment, biological pretreatment with enzymes has extensive research potential given the advantages of low energy consumption, environmental friendliness and lower production cost. Different lignocellulosic biomasses need different types of pretreatments because the structural features of cellulose play important roles in enzyme hydrolysis that affect outcomes such as polymerization degree, cellulose crystallization arrangement, surface area accessibility, particles size, and the existence of hemicelluloses and lignin (Bhatia et al., 2020; Sankaran et al., 2020). Our results showed that *Tl*SWO directly alters the cellulose structure, which in turn increases its hydrolysis rate. Using optimized preconditioning and molecular design, *Tl*SWO could be a promising additive for improving lignocellulosic biomass generation performance.

## Data Availability Statement

The datasets presented in this study can be found in online repositories. The names of the repository/repositories and accession number(s) can be found below: https://www.ncbi.nlm.nih.gov/genbank/, XP_747748; https://www.ncbi.nlm.nih.gov/genbank/, ACH57439; https://www.ncbi.nlm.nih.gov/genbank/, ADZ74267; https://www.ncbi.nlm.nih.gov/genbank/, CAB92328; and https://www.ncbi.nlm.nih.gov/genbank/, ABV57767.

## Author Contributions

HZ and YW performed the experiments. FZ and RB designed and performed the synergism experiments and analyzed the data. BY and XX designed the research and participated in the bioinformatics analysis. FZ and HL revised the manuscript. All authors read and approved the final manuscript.

## Conflict of Interest

The authors declare that the research was conducted in the absence of any commercial or financial relationships that could be construed as a potential conflict of interest.

## Footnotes

1 https://exed.biocatnet.de

2 http://www.ncbi.nlm.nih.gov/BLAST

3 http://genes.mit.edu/GENSCAN.html

4 http://www.cbs.dtu.dk/services/SignalP

5 http://www.cbs.dtu.dk/services/NetNGlyc

6 http://www.expasy.org/tools

7 http://br.expasy.org/prosite

8 http://clavius.bc.edu/∼clotelab/DiANNA

## Abbreviations

CBM: carbohydrate binding module
CMC-Na: carboxymethylcellulose sodium
CNC: cellulose nanocrystals
GH: glycoside hydrolase
PASC: phosphoric acid swollen cellulose
PCS: pre-treated corn stover.
