# Supplementary material for: A Swollenin From Talaromyces leycettanus JCM12802 Enhances Cellulase Hydrolysis Toward Various Substrates
Source: Front Microbiol. 2021 Mar 29;12:658096. doi: 10.3389/fmicb.2021.658096 (PMC8039133; doi:10.3389/fmicb.2021.658096)
Supplement: Supplementary file 1 [file Table_1.DOCX]

***Supplementary Material***

**
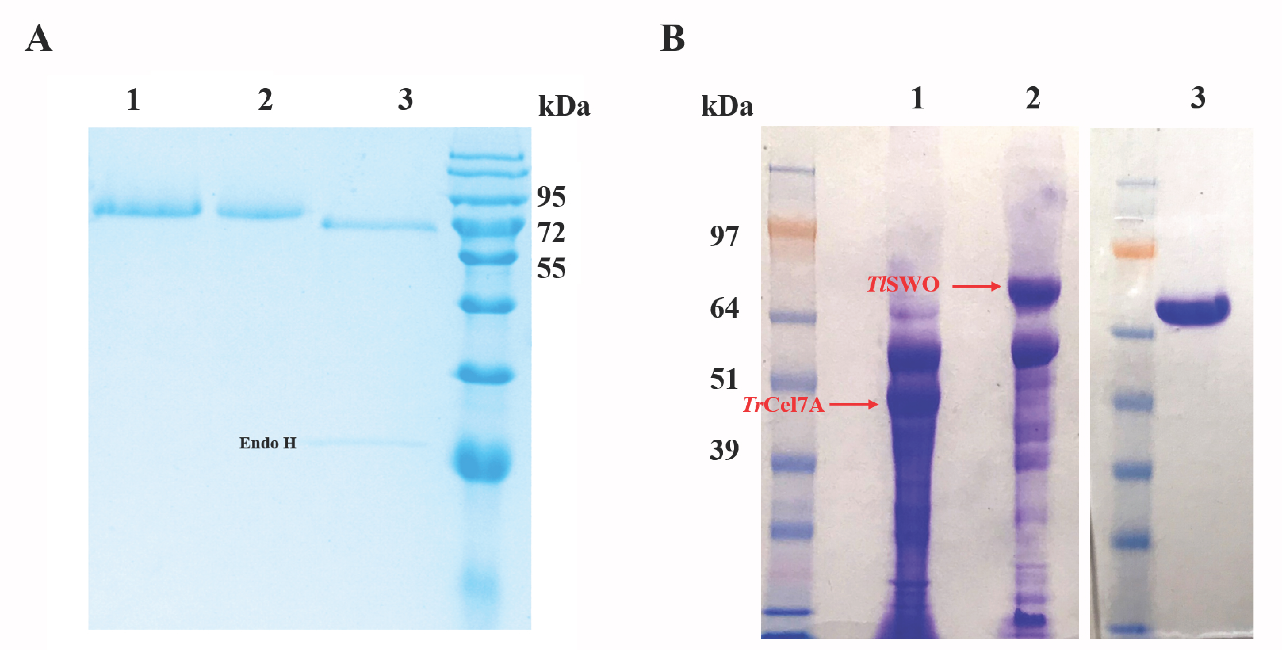
**

**Supplementary Figure 1.** Production and purification of recombinant *Tl*SWO in *Pichia pastoris* and *Trichoderma reesei*. A: SDS-PAGE analysis of the culture supernatant of *Tl*SWO produced by *Pichia pastoris*. Lane 1: the crude enzyme of *Tl*SWO; lane 2, the purified *Tl*SWO; lane 3, the deglycosylated *Tl*SWO; B: SDS-PAGE analysis of the culture supernatant of *Tl*SWO produced by *Trichoderma reesei*. Lane 1: the crude enzyme of positive control contained *Tr*Cel7; lane 2, the crude enzyme of *Tl*SWO; lane 3, the purified *Tl*SWO.

**
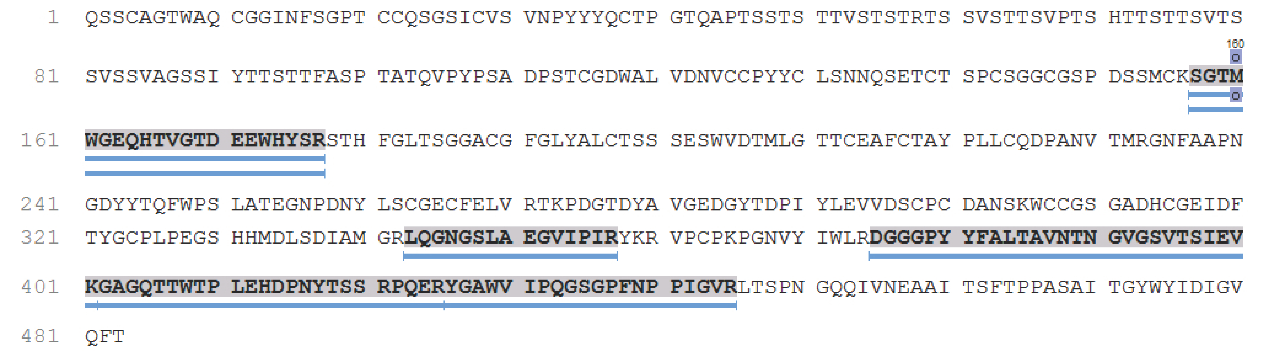
**

**Supplementary Figure 2.** MALDI-TOF-MS analysis of the recombinant protein *Tl*SWO.

**
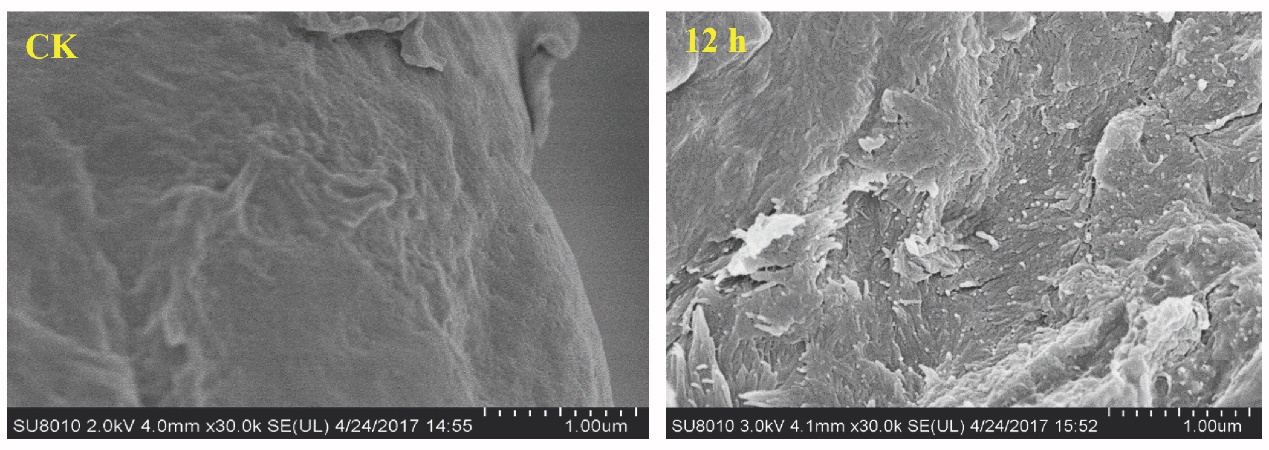
**

**Supplementary Figure 3.** Scanning electron microscopic analyses of Avicel. Ten milligrams of Avicel was incubated with 300 μg of purified *Tl*SWO in 100 mM citric acid-Na_2_HPO_4_ buffer (pH 4.0) for 12 hours.
